# Transnational Families and the Well-Being of Children: Conceptual and Methodological Challenges

**DOI:** 10.1111/j.1741-3737.2011.00840.x

**Published:** 2011-08

**Authors:** Valentina Mazzucato, Djamila Schans

**Affiliations:** Maastricht University

**Keywords:** child-care arrangements, child fostering, children, migrant families, transnational families

Family research and scholarship on immigrant families has evolved in the past decade to include factors such as community context, family environment, and individual attitudes to explain immigrant family formation and functioning (see Glick, 2010, for a review). Nevertheless, methodologically and theoretically, families are still predominantly conceived of as nuclear, living together, and bounded by the nation state. Family studies emphasize geographical proximity as a prerequisite for interaction and exchange within families, thereby eliding family ties that cross national borders. As a result, family practices across borders are ignored or assumed to be unfeasible (Baldassar & Baldock, 1999; Mazzucato & Schans, 2008) and transnational families—conceived of as families with members living in different nation states—have been treated as a temporary phenomenon, with family reunification in the host society as the preferred outcome for all family members (Landolt & Da, 2005).

In contrast, research from the past decade in the areas of migration and development has demonstrated that individuals' migration-related choices are related to their family members' needs and that migration affects migrants' origin countries. This area of research, however, largely focuses on the economic effects of remittances on households as a whole and rarely analyzes the differential impact of remittances on individual family members (Adams & Page, 2005; Ratha, 2003). Moreover, studies from this field do not consider noneconomic effects such as the impact of migration on the well-being of family members who live apart.

In this special section, we broaden analyses in the fields of both family and migration studies by examining the effects that migration has on the well-being (defined as psychological, educational, and health outcomes) of children who are left in the country of origin. Here, we use the word children to emphasize the relationship between a young person and his or her parent or caregiver; however, the contributions to this special section examine children and youths up to 18 years of age.

Transnational family arrangements are prevalent worldwide because of stringent migration policies in migrant receiving countries that make it difficult for families to migrate together, families' attempts to escape violent conflict or persecution, or family members' preferences, especially in societies where child fostering is a common practice, such as in many places in Africa. The exact prevalence of transnational family arrangements is unknown, however, because of a scarcity of quantitative evidence caused by the lack of academic and policy attention to this phenomenon. Reports by nongovernmental organizations and international organizations such as Save the Children and UNICEF indicate that approximately 25% of children in selected migrant-sending countries have at least one parent abroad. This estimate appears sufficiently large to justify further research on transnational families and the well-being of children. As a consequence, scholars have begun to turn their attention toward transnational families, though most of the scholarship thus far is in the form of qualitative case studies.

This introduction begins with an overview of current scholarship on transnational families from different disciplinary backgrounds and identifies important contributions to the literature and gaps in our knowledge. In the next section, we highlight three conceptual and methodological challenges and discuss how the articles in this special section advance our understanding of transnational families and the well-being of children. We conclude by discussing important elements of an agenda for future research on transnational families in general and their impact on child well-being in particular.

## Current Scholarship

In the last decade, studies on transnational families have emerged in which scholars from different disciplines have engaged with the topic of families with members who live across national borders and the effects of such transnational living arrangements on children (Borraz, 2005; Dreby, 2007; Parrenas, 2005; Schmalzbauer, 2008). These studies have shown that whether children benefit from their parents' migration depends not only on the outcomes that are studied (economic vs. psychological outcomes) but also on the characteristics of the parent and child. Children might benefit from remittances while suffering emotionally from prolonged separation (Borraz; Dreby; Heymann et al., 2009; Kandel & Kao, 2000; Lahaie, Hayes, Markham Piper, & Heymann, 2009; Suarez-Orozco, Todorova, & Louie, 2002; Schmalzbauer, 2004). Moreover, these economic and psychological processes are gendered (Aranda, 2003). For example, Abrego (2009) found that families in which the mother migrated are more likely to thrive economically than father-migrant families because of the extreme sacrifices mothers make to send remittances home. However, Parrenas found that children experience more emotional problems when their mother migrates compared to when their father migrates because of traditional gender norms related to care.

The role of the caregiver of the child in the country of origin is understudied, but the scant studies on the topic suggest that the caregiver is extremely important for the well-being of the child. Lahaie et al. (2009, p. 308) showed that children who take care of themselves (self-care) are three times as likely to experience behavioral and academic problems as children in other care arrangements. In a study by Dreby (2007), children felt abandoned by their parents and in some cases responded by detaching themselves from the parent that left. Such feelings might lead to unwanted behavior such as quitting school or gang involvement. Thus, for migrants who left to ensure better opportunities for their children, the unintended consequences of their migration might include a strained relationship with their children and a loss of educational opportunities for their children.

The aforementioned studies show that some exciting work has recently emerged in the field of transnational families and child well-being. Indeed, as Glick (2010, p. 507) mentioned in her recent review of research on immigrant families, “[r]esearchers have become increasingly aware of the bi-national realms in which many immigrant families operate and the strategies they employ.”

Despite this progress, some significant gaps remain in scholarship on transnational families. First, most studies in this area are small-scale qualitative studies that do not collect systematic data on the topic of child well-being. It is therefore difficult to assess and verify the information presented in these studies. The second gap is related to a more general criticism of studies on transnationalism: “They study cases of the phenomenon itself so it is difficult to say anything about the extent of the phenomenon and whether it is increasing” (Portes, 2001). There is usually no comparison group of children in nonmigrant families or of children who migrated together with their parents, which makes it impossible to determine whether the observed phenomena are particular to transnational families or affect a wider group of people than just those in families that live across borders. Moreover, most data are collected at one end of the transnational spectrum, with only the parents in the host country or only the caregivers and children in the country of origin as subjects (Mazzucato, 2008). In addition, children are mostly not interviewed themselves; rather, their caregivers are asked to evaluate the well-being of the child. Finally, the majority of studies on the well-being of children who are left behind have focused on one particular stream of migration: from Latin America (specifically Mexico) to the United States. This focus ignores increasing trends in transnational families in other migrant sending areas such as Sub-Saharan Africa and parts of Asia. With the exception of the Philippines, however, little is known about the effects on children in these parts of the world (see Huang, Yeoh, & Lam 2008, for a special issue on Asian transnational families).

The contributions in this special section are the result of a workshop organized by Mazzucato to address the gaps identified above, entitled “Researching Transnational Families, Children and the Migration-Development Nexus,” held at the University of Amsterdam in the Netherlands in December 2008. The workshop brought together experts from various disciplines who have approached these issues from diverse perspectives using various methodologies. The articles in this special section were written by authors who conduct cutting-edge research on the topic of transnational families and children within the disciplines of family sociology, anthropology, demography and geography (Bernardi, 2011; Bledsoe & Sow, 2011; Donato & Duncan, 2011; Graham & Jordan, 2011; Nobles, 2011). Below, we discuss the methodological and conceptual challenges we have identified and address how the articles in the special section help overcome some of these challenges.

## Discussion

The gaps in current scholarship on transnational families are caused by methodological and conceptual challenges that are particular to the study of families across borders. These challenges are threefold: conceptualizing families across borders and the consequent need for multisited research designs, taking cultural norms of family into account and the consequent need to incorporate anthropological insights about migrants' origin cultures, and moving beyond the nuclear family and the consequent need to incorporate new units of analysis into research.

### Conceptualizing Families Across Borders and Multi-sited Research Designs

Transnational families, like all transnational phenomena, encompass multiple national contexts. This presents a challenge to research on migration that had, until the turn of the 21st century, been predominantly characterized by methodological nationalism, that is, research in which the units of analysis were located in one nation state (Wimmer & Glick Schiller, 2002). As discussed above, the dominant focus of research on the family has been on families living in one location, and in cases where transnational families were examined, data were collected mostly in just one nation state. Even transnational scholarship, which has highlighted the importance of taking both migrant sending and receiving countries into consideration, focuses predominantly on either one context or the other (Mazzucato, 2008). This leaves part of the family out of focus and provides researchers with only a partial view of the factors that impact children's well-being.

To advance scholarship on transnational families, multisited research and data collection in different nation states are necessary. Scant data of this type are available, however. Qualitative studies that collect data from different family members in binational settings (e.g., Dreby, 2007; Schmalzbauer, 2004) are a step in this direction, but to systematically analyze the effects of transnational family arrangements, data on relevant comparison groups are also needed. Donato and Duncan (2011) fill a gap in the literature by comparing health outcomes for children in three types of families: children living with their migrant parents in the United States, children living with parents who migrated and returned to Mexico, and children who live in Mexico with parents who never migrated. Their finding of different outcomes for each of these groups of children highlights the importance of both collecting binational data and including nonmigrants and return migrants as comparison groups in studies on transnational families.

### Cultural Context and Norms About Family

Different cultural norms regarding the family exist in different places around the world. This means that the nuclear family cannot be assumed to be the best option for all families. It is important to recognize that not all families that are separated by borders have family reunification as their ultimate goal. Transnational family life may not necessarily lead to family disintegration and may be part of a strategy for social mobility for all members (Olwig, 2002). Furthermore, transnational family forms may be the preferred option for migrant parents depending on their child raising norms (Bledsoe & Sow, 2011).

A balanced focus on both sending and receiving countries allows the researcher to gain a thorough understanding of the institutions and cultural norms that guide family relationships in the migrant sending country. This understanding is lacking in studies that mainly assume a Western nuclear family model without explaining the culturally relevant notions of family that influence family relationships in the particular case under study (Mazzucato, forthcoming).

Bledsoe and Sow's (2011) exploratory paper on the phenomenon of children of West African immigrants in Europe and North America being sent back to their home country offers new insights into the dynamics of transnational child raising. Disciplinary problems at home and in school in the host country can lead parents to send their children back to the country of origin to reconnect them to their culture and family, even if this means that they will be separated from their parents for an extended period of time. A better understanding of socialization norms may help explain why parents sometimes leave their children behind or even send them back to the country of origin to be raised.

Cross-country comparisons are largely missing in transnational migration studies, even though such comparisons would provide useful means to understand the effect of different family norms on child well-being. Graham and Jordan (2011) address this gap when they compare the psychological well-being of children left behind in four countries in Southeast Asia. Their findings show that although children of migrant fathers in Indonesia and Thailand are more likely to have poor psychological health compared to children in nonmigrant households, this is not the case for children in the Philippines or Vietnam. These findings emphasize the point that context must be taken into account when comparing the effects of migration on child well-being around the globe.

### Beyond the Nuclear Family: Incorporating New Actors and New Units

One method proposed by Marcus (1995) to move beyond methodological nationalism is to follow the people. In the case of transnational families, this means following the various family members to where they are located: where migrants live and where their family members reside. This enables researchers to understand the effects of transnational family life on all family members. This method has been applied in some of the studies reviewed above, especially those in the fields of sociology of the family and gender studies, but the focus of these studies has been primarily on only a few of the family members, namely, mothers and children.

To get a complete picture of transnational family life, it is important to add fathers to the picture, not just on the sideline but as main actors. Nobles (2011) looks at the consequences of residential separation for children in Mexico whose fathers migrated to the United States. Although parent–child separation has long been a central theme in family research, it has typically been framed in terms of divorce rather than as a consequence of international migration. Therefore, even though it is well known that the composition and stability of children's living arrangements are predictors of children's well-being, these factors are rarely studied in migration-focused research, especially not using quantitative methods. Nobles overcomes this gap in the literature by using representative data to compare the effects of father–child separation caused by divorce and by migration and finds that these forms of separation are distinct experiences from the perspectives of children. Further, Nobles finds that levels of interaction with non-resident fathers are higher when the separation is caused by migration instead of divorce, regardless of the considerable geographic separation that migration entails. Moreover, ties with migrant fathers are positively correlated with schooling outcomes.

Other family members who are important for children's well-being in transnational families are caregivers. New actors take on caregiving tasks when biological parents and children do not live in the same country. Caregivers may be kin relations, such as the child's maternal grandmother or the child's paternal aunt. There are also instances of non-kin relations taking care of migrants' children, such as pastors, good friends, or fellow churchgoers. This implies that new units of analysis must extend beyond the nuclear family to include these new actors.

Bernardi (2011) offers methodological insights for the study of transnational families through the analysis of social networks related to care. She argues that a mixed-methods research design in which both quantitative and qualitative approaches are used to assess children's networks and their functioning offers several advantages for the area of child well-being in transnational families. Quantitative network analyses aim to reconstruct the potential and actual relational support that is available to children in a context where interactions may be hindered by temporary or prolonged periods of separation. Qualitative analyses can address family strategies to maintain relationships across legal borders and geographical distance. The use of networks allows the inclusion of units that are larger than the nuclear family, which is particularly relevant when the extended family is the dominant institution through which caring is given and received.

## Conclusion

The articles in this special section make important advances in the study of transnational families in general and of the well-being of children in particular. All of the contributions address at least one of the methodological and conceptual challenges we have identified: that families are not bounded by the nation state; that cultural norms around the family are important to understanding transnational families' choices, the forms they take, and the effects they experience; and that it is necessary to include more actors than just the nuclear family. Bledsoe and Sow (2011) show that family reunification is not necessarily the end of transnational family life and that dynamics within and outside the family can lead parents to send their children back to their country of origin. Nobles includes fathers in her study of transnational families—an area that typically focuses on mothers. Graham and Jordan are among the first scholars to have collected data on the well-being of children who are left behind in an international comparative design, and Donato and Duncan compare outcomes for migrant children and children who are left behind with nonmigrant children to be able to make more rigorous claims about the effect of transnational family life. Finally, Bernardi argues the importance of studying the extended networks of transnational nuclear families using both quantitative and qualitative research methods. Although all of the articles contribute to filling several of the gaps we identified, none tackles them all, and some challenges have only begun to be addressed. Thus, we propose here important elements that we believe should be included in an agenda for future research on transnational families.

### Cultural Contextualizations: Shifting Norms and Institutions

Earlier, we argued that it is important to embed studies of transnational families in cultural understandings of the norms that guide family life. It is also important, however, to recognize that changes in these norms occur as a result of migration. The study of child fostering systems is a case in point. Child fostering systems have been studied by anthropologists, especially in the West African region, where the system is an age-old institution (Allman 1997; Bledsoe & Isiugo-Abanihe 1989; Goody 1982; Isiugo-Abanihe 1985; Page 1989; Schildkrout 1973; Shell-Duncan 1994). These studies give attention to different actors in child fostering systems, but, thus far, most studies have focused on the “traditional” system of fostering children between rural and urban areas of the same country. In this system, parents either migrate to cities for work and entrust their children to extended family members in rural villages or they send their child to extended family in the city in the hope that a brighter future will await the child in an urban context. In this case, parents are often poor rural dwellers who foster their children with family in the city who use the children as household help (Goody). These studies show that fostering arrangements are based on informal agreements about the rights and responsibilities of each of the actors. These rights and responsibilities are subject to negotiation and are based on reciprocal relations and trust between extended family members (Alber, 2003; Goody; Isaac & Conrad, 1982).

Long-distance, international migration poses some interesting questions for the study of child fostering because many of the conditions that prevail in traditional fostering are not present. First, geographic distance and travel costs make it difficult for parents and caregivers to maintain relationships of trust through regular visits. Second, caregivers tend to live in urban contexts where they cannot rely on the help of extended family with the tasks of childrearing, as is done in a rural setting. Further, migrant parents do not want their children to be used for household help, but instead want them to receive a high-quality education. Third, migrant parents are perceived as having more economic resources than caregivers and therefore are expected to provide financial help to the caregiver and his or her family. Research on transnational families can contribute to anthropological studies on child fostering by considering how local institutions that guide family norms change as a result of modern conditions of international migration (see Leinaweaver, 2010, as an example).

### Impact of Laws and Policies

To date, there is limited knowledge about how migration status affects transnational family practices. Recent qualitative research (Bernhard, Landolt, & Goldring, 2008; Fresnoza-Flot, 2009) indicates that undocumented migrant mothers in France and Canada face challenges in their role as transnational mothers because of their migration status. Unable to visit family back home or to bring children to visit, they have to deal with separations of unexpected length and with feelings of tension and guilt. Access to documents and citizenship is restricted in different ways among nation states and differs by national origin within a country. The effects of parents' migration status on both parents and children should be studied further in comparative research. The children of undocumented parents who are left behind in the country of origin might be unable to see their parents for extended periods of time, and parents' undocumented status may also affect the amount of money or goods they are able to send. If undocumented parents find a way to bring their children to the host country, children may face obstacles to obtaining adequate education and health care and may become undocumented workers themselves.

Including migration status in the analysis of transnational family dynamics will only become more important as migration policies in most countries become stricter. In recent years, family reunification has become more difficult, expensive, and time-consuming in nearly all Western migrant-receiving countries. As Bernhard et al. (2008, p. 25) showed, the original decision to leave a child behind reflects the options that are available to a family in their context of departure. The factors that result in a prolonged separation or a failed reunification, however, are largely the result of the context at reception, including the migration policies that impinge on family migration and reunification. Bledsoe and Sow (2011) argue that, beyond policy, the ethnic and racial attitudes of the people in the host societies will influence parental decisions regarding where to raise their children.

Policies in both the sending and receiving countries are ill equipped to confront the difficulties associated with transnational family arrangements. Indeed, migration policies create transnational families by limiting opportunities for family migration. Recent policy changes have made reunification more problematic and increased periods of separation between parents and children. Longer periods of separation might in turn lead to more problems associated with reunification for both parents and children, even though social service agencies often see reunification as the end of families' need for services and support (Bernhard et al., 2008). In migrant-sending countries, almost no policies exist that target children who are left behind by their parents (Yeoh & Lam, 2006).

### Mixed Methods, Multiple Sites, Matched Samples, and International Comparisons

The most insightful studies in the field of transnational families are those that integrate large-scale quantitative methods with in-depth qualitative understandings of how relationships function. The integration of methods is challenging, however, and even when both methods are used, findings often draw mainly from one part of the study or the other, with little integration of findings.

We argue that although an emerging literature on transnational families has made critical contributions in terms of raising the issues encountered by such families, there is a need to collect more systematic data on transnational families to understand the extent of these issues and their effects on the various members involved. A number of important research questions have yet to be addressed, for example: How pervasive are transnational families? What effects do they have on all of the actors, not only children, and how do the effects and issues that affect transnational families differ from those of families who live together?

Another purpose of systematic analysis on transnational families is to distinguish between different types of transnational families. This is important because, although there are many different types of transnational childrearing arrangements, none of the studies reviewed above categorizes these types in a systematic manner. Childrearing arrangements can take different forms, including those in which children are raised by a caregiver in the extended family, those in which children are raised by their biological mother or father, those in which children are raised by a non-kin caregiver, and those in which children take care of themselves. It is therefore important to ask: What are the different types of transnational childrearing arrangements and do they have differential impacts on the various actors? Finally, there is a need for longitudinal data collection that follows members of transnational families over time to overcome issues of selectivity in the analysis of the effects of transnational lifestyles and of how decisions concerning whether to live separately or to reunify are shaped by changing contexts both at home and abroad.

There are some existing studies that combine mixed methods and matched samples over multiple sites. Massey's (1987) ethnosurvey methodology that mixed in-depth anthropological work with large-scale surveys is of interest, although the component in which migrants are matched to people back home was not conducted at the scale that was originally intended. This is the precise component that is necessary for the study of transnational families, their relationships, and resources that cross national borders. A number of studies use matched sample methodologies (with medium-sized samples of around 150 people), which are especially suited to the study of transnational families because they sample individuals who are connected across multiple sites. This methodology has been used by Osili (2004), who studied remittance behavior for housing construction, Dreby (2007), who studied family relationships between Mexican migrants and their children back home, and Schmalzbauer (2004), for the case of Honduran migrants and their family members. Mazzucato (2008) added a simultaneous component to the method by using a team of researchers to study a matched sample of people *at the same time*. This enabled the study of the small, everyday actions and transactions that influence how transnational relationships take shape but that often go unobserved when single researchers visit multiple sites sequentially and need to rely on respondent recall.

The further development of such methodologies can improve research on transnational families and broaden our understanding of the contributions of migration to the development of migrant sending countries and especially to migrants' families. Two current projects, Transnational Child Raising Arrangements (TCRA) and Transnational Child Raising Arrangements between Africa and Europe, which are funded by the Netherlands Organisation for Scientific Research and NORFACE, respectively, use teams of researchers from different migrant-sending and -receiving countries in Europe and Africa to study the effects of transnational families on the different members involved in transnational care arrangements. They study matched samples of parents, caregivers, and children across nations and use mixed methods to measure the various actors' outcomes and study local institutions in both sending and receiving contexts to situate the results within cultural understandings of “the family.” The projects integrate both quantitative and qualitative data on migrant parents, children who are left behind, and caregivers in several European and African countries to allow for cross-country comparisons. They measure multiple outcomes related to education, job performance, health, and emotional well-being, thus acknowledging that migration impacts various realms, not just the economic realm. The projects also include a focus on the institutions that affect how TCRAs function, such as schools in the country of origin and migration laws in the destination country. They pay particular attention to the role played by norms around family, upbringing, and intergenerational relationships in general in the analysis of the effects of TCRAs on the different actors. Such collaborative projects by teams of researchers who collect large-scale and longitudinal data through mixed methods is a step toward filling the gaps identified in this special section.
